# Poliovirus seroprevalence before and after interruption of poliovirus transmission in Kano State, Nigeria

**DOI:** 10.1016/j.vaccine.2016.08.058

**Published:** 2016-09-30

**Authors:** Zubairu Iliyasu, Harish Verma, Kehinde T. Craig, Eric Nwaze, Amina Ahmad-Shehu, Binta Wudil Jibir, Garba Dayyabu Gwarzo, Auwalu U. Gajida, William C. Weldon, M. Steven Oberste, Marina Takane, Pascal Mkanda, Ado J.G. Muhammad, Roland W. Sutter

**Affiliations:** a Department of Community Medicine, Aminu Kano Teaching Hospital & Bayero University Kano, Nigeria; b World Health Organization, Geneva, Switzerland; c World Health Organization, Abuja, Nigeria; d National Primary Health Care Development Agency, Abuja, Nigeria; e Department of Pediatrics, Murtala Mohammed Specialist Hospital, Kano, Nigeria; f Department of Pediatrics, Aminu Kano Teaching Hospital & Bayero University, Kano, Nigeria; g Centers for Disease Control and Prevention, Atlanta, GA, United States

**Keywords:** Poliomyelitis, Seroprevalence, Kano, Nigeria, Oral poliovirus vaccine

## Abstract

**Introduction:**

In September 2015, Nigeria was removed from the list of polio-endemic countries after more than 12 months had passed since the detection of last wild poliovirus case in the country on 24 July 2014. We are presenting here a report of two polio seroprevalence surveys conducted in September 2013 and October 2014, respectively, in the Kano state of northern Nigeria.

**Methods:**

Health facility based seroprevalence surveys were undertaken at Murtala Mohammad Specialist Hospital, Kano. Parents or guardians of children aged 6–9 months, 36–47 months, 5–9 years and 10–14 years in 2013 and 6–9 months and 19–22 months (corresponding to 6–9 months range at the time of 2013 survey) in 2014 presenting to the outpatient department, were approached for participation, screened for eligibility and asked to provide informed consent. A questionnaire was administered and a blood sample collected for polio neutralization assay.

**Results:**

Among subjects aged 6–9 months in the 2013 survey, seroprevalence was 58% (95% confidence interval [CI] 51–66%) to poliovirus type 1, 42% (95% CI 34–50%) to poliovirus type 2, and 52% (95% CI 44–60%) to poliovirus type 3. Among children 36–47 months and older, seroprevalence was 85% or higher for all three serotypes. In 2014, seroprevalence in 6–9 month infants was 72% (95% CI 65–79%) for type 1, 59% (95% CI 52–66%) for type 2, and 65% (95% CI 57–72%) for type 3 and in 19–22 months, 80% (95% CI 74–85%), 57% (49–63%) and 78% (71–83%) respectively. Seroprevalence was positively associated with history of increasing oral poliovirus vaccine doses.

**Conclusions:**

There was significant improvement in seroprevalence in 2014 over the 2013 levels indicating a positive impact of recent programmatic interventions. However the continued low seroprevalence in 6–9 month age is a concern and calls for improved immunization efforts to sustain the polio-free Nigeria.

## Highlights

• Polio seroprevalence surveys help measure progress towards polio eradication.
• Nigeria program conducted multiple seroprevalence surveys in northern states.
• This article covers seroprevalence survey in Kano Nigeria in 2013 and 2014.
• Data represents levels before and after the interruption of poliovirus transmission.
• Significant improvement in seroprevalence in 2014 over 2013, but gaps continue.
• Good participation even by vaccine refusers in this health facility based project.

## 1 Introduction

Since the World Health Assembly resolved in 1988 to eradicate poliomyelitis globally [1], great strides have been made towards achieving this goal. The overall incidence of poliomyelitis has declined by more than 99% [2], wild poliovirus (WPV) type 2 has been certified eradicated globally [3], and more than three years have passed since the last case of WPV type 3 was detected in November 2012 in Nigeria [2], [4].

Nigeria has been removed from the list of polio endemic countries by World Health Organization (WHO) after more than 12 months having passed since the detection of the last case of WPV type 1 in the country having onset of paralysis on 24 July 2014 [5]. Only two countries, Pakistan and Afghanistan remain endemic for WPV type 1. The main challenges to eradicating poliomyelitis in the remaining endemic countries include limited access to children in security compromised areas and inadequate service delivery of oral poliovirus vaccine (OPV) [6], [7].

Nigeria has been polio-endemic all along and achieved the milestone of interrupting poliovirus transmission after a long fought battle. Historically, Nigeria has been reporting a large proportion of global polio case burden. A consistent downward trend in the number of confirmed WPV cases started in 2012, leading to the current polio-free status. As per WHO data (as of 05 May 2016), Nigeria had 53 cases of confirmed WPV1 in 9 States in 2013, 6 cases in 2 States in 2014, and no cases in 2015 [8]. Recent detection of WPV1 cases in Borno state is under investigation.

Besides WPV transmission, Nigeria has the problem of persistent transmission of circulating vaccine-derived poliovirus type 2 (cVDPV2) [9], [10], [11]. Cases of cVDPV2 detected increased from 4 in 2013 to 30 in 2014. Bivalent (types 1 and 3) OPV (bOPV) was used in most Supplementary Immunization Activities (SIAs) covering the 11 high-risk northern Nigerian states during this period. The associated decline in type 2 immunity is likely to have caused the increase in cVDPV2 cases in 2014. However, a number of trivalent OPV (tOPV) SIA rounds conducted since August 2014, as well as targeted campaigns with inactivated polio vaccine (IPV), are likely to have improved levels of immunity to type 2 poliovirus and subsequent decline in cVDPV2. In 2015, one case of cVDPV2 was detected in the country in the Federal Capital Territory (FCT) Abuja and this year in 2016, one case of VDPV2 has been reported from Jigawa state and one environmental isolation from Borno state of Nigeria.

Poliovirus transmission in Nigeria in the last two years preceding the last case has largely been confined to North West and North East zones, and most cases having been reported from Kano, Borno and the Yobe states. Kano State, the most populous in northern Nigeria with 44 LGAs, has had the most intractable transmission of poliovirus in the region. Even when there was a persistent decline in the number of polio cases in Nigeria, Kano reported a relatively large number of cases: 15 WPV1 cases in 2013 and 5 WPV1 and 10 cVDPV2 cases in 2014. Within Kano, Kano Metropolitan Area (KMA), the urban area comprising of 8 very high risk local government areas (LGAs), has always been categorized as very high risk for polio.

We conducted seroprevalence surveys in KMA to measure polio seroprevalence levels in 2013 and then repeated in 2014 in the different age groups of interest. In addition, we evaluated factors probably associated with seroprevalence levels. The data were intended for assessment of program performance and to guide future actions. An earlier seroprevalence carried out in this area in 2011 was published in *Vaccine* [12], and preliminary data summarized in JID supplement in 2016 [13].

## 2 Methods

### 2.1 Study design and its rationale

A health facility-based cross-sectional design was used in both the surveys. Experience of 2011 survey had shown that the sample representation from 8 LGAs of KMA was quite proportionate to the population. Both the vaccine acceptors and refusers had participated. Moreover, health facility based was an operational advantage in this area with considerable security risk for field operations.

Seroprevalence levels were assessed in four age groups in the 2013 survey: 6–9 months, 36–47 months, 5–9 years, and 10–14 years. Seroprevalence was found to be quite low in 6–9 month group, possibly attributed to a dip in the quality of SIAs due to killing of polio vaccinators in Kano during that period [14]. So it was decided to do a follow-up survey in 2014 in 6–9 month and 19–22 month age groups, the latter corresponding to 6–9 month cohort at the time of 2013 survey.

### 2.2 Objectives of 2014 survey were

(1) To assess seroprevalence in 6–9 month olds and compare it with levels in 2013, as an indicator of recent program quality.
(2) To demonstrate improvement in seroprevalence in 19–22 month olds, birth cohort that was aged 6–9 months at the time of 2013 survey.

### 2.3 Selection of the study area

Kano state and KMA within the state were selected for assessment of seroprevalence primarily due to the high incidence of poliomyelitis cases in recent years. KMA comprises eight local government areas (LGAs): Kano Municipal, Fagge, Nassarawa, Dala, Gwale, Tarauni, Ungogo, and Kumbotso. The location of Kano state and KMA are indicated in the map of northern Nigeria, see previous publication 12.

### 2.4 Selection of study site

Murtala Mohammad Specialist Hospital (MMSH) was selected as the study site because it is the largest hospital in KMA. This public sector hospital has a very high turnover of patients in the pediatrics outpatient department (OPD). Patients come from a wide catchment area including our area of interest i.e. the 8 LGAs of KMA, with a total population of around 3.7 million.

### 2.5 Sample size

For the 2013 survey, the actual seroprevalence from the 2011 survey was used for sample size calculations. A total of 600 children were required to be enrolled: 150 from each of the four age groups. For the 2014 follow-up survey, due to much lower seroprevalence in 2013, a somewhat larger sample size was needed to maintain the same precision of ±7.5% and 95% confidence. Using the type 3 seroprevalence for the 6–9 month age group of 51% from the 2013 survey, sample size of 171 was arrived at. It was inflated to 180 per age group to account for withdrawal and inability to draw sufficient quantity of blood, the total sample size used for two age groups was 360.

### 2.6 Eligibility criteria

Infants fulfilling age requirement and residing for at least one month in KMA with consenting care-givers were eligible to participate, except those with (a) contraindication to venipuncture; (b) serious acute illness requiring hospitalization; or (c) diagnosed or suspected congenital immunodeficiency disorder in the subject or an immediate family member.

### 2.7 Enrollment and survey procedures

Enrollment for the 2013 survey was done between 12 September and 2 October. Corresponding period for the 2014 survey was 15 October to 4 November. Protocols for enrollment and survey procedures were similar for the two surveys. Parents and guardians of patients visiting the pediatric OPD of MMSH were approached for participation of their infant/child in this survey. Initial screening for age and area of residence was done by a study staff in the OPD. The physician in the study clinic explained the study to the parents or guardians and assessed children for inclusion criteria. After obtaining informed consent of caretakers and assent from older children, weight and length/height were measured, immunization history was taken and the questionnaire was completed. Regulations governing research involving human subjects were followed throughout the course of the study.

### 2.8 History of Routine and SIA doses

As per Nigeria’s national policy, routine immunization (RI) doses of tOPV (switched to bOPV since April 2016) were recommended to be administered at birth, 6, 10 and 14 weeks, and multiple SIAs were implemented in the Kano area every year with bOPV or tOPV, wherein all children up to 5 years old were expected to receive an additional dose every time irrespective of their immunization status. As a result, the study participants were eligible for a maximum of 4 RI doses and a variable number of SIA doses depending on the age of the child and completeness of coverage in SIA. Overall going by the SIA calendar, 6–9 month old infants could have received a maximum of 6–8 bOPV doses through SIAs since birth in 2013 survey and 6 bOPV doses and 1 tOPV dose in 2014 survey.

### 2.9 Blood collection and antibody testing procedures

One milliliter of blood was collected through venipuncture. Sera were shipped to the Centers for Disease Control and Prevention (CDC) in Atlanta. Sera were tested in triplicate for levels of neutralizing antibody titers against poliovirus types 1, 2, and 3, respectively, using modified micro-neutralization assays, using dilutions of 1:8–1:1024 [15], [16]. For seroprevalence, antibody titers ⩾1:8 were regarded as positive (i.e., detectable titer).

### 2.10 Definitions

History of doses received by the study participants was primarily based on verbal recall because immunization card/other documentary evidence were not available with most parents. RI and SIA doses were recorded separately and added to arrive at the total doses. Doses were included regardless of how close they were in time to other doses except when the last dose was on the day of blood sample collection. Nutritional status was measured using height and weight. Data were compared to the standard distribution of a reference population using WHO Anthro software [17]. The reference population was the WHO Child Growth Standards based on the WHO Multicenter Growth Reference Study (MGRS) [18]. Categories of malnutrition were based on standard deviation (SD) units (*z*-scores) below the mean of the reference population. These categories were defined as normal (<2 SD), severe to moderate (2.0–2.99 SD) and extreme (⩾3 SD).

### 2.11 Data management and statistical analysis

Questionnaires were double-entered into an initial database using CSPro software version 5.0 [19]. Data analysis was done using R (R Foundation) versions 3.0.2 (2013) and 3.1.1 (2014) [20]. Chi-square tests were used to assess the association between dichotomous predictors and seroprevalence. The Cochrane-Armitage test for trend was used to test for trend in seroprevalence across sub-groups of ordinal variables. Logistic regression was used to estimate adjusted odds ratios and to assess the association of other risk factors (such as gender, education level, and nutrition) with seroprevalence by the number of OPV doses received in infants 6–9 months of age. All risk factors were considered in the initial models, and covariates with type III p-value <0.10 were included in the final models.

## 3 Results

### 3.1 Final study sample and distribution

The final sample consisted of 602 subjects for the 2013 survey (4 age groups) and 363 subjects for the 2014 survey (2 age groups). Subjects from all eight LGAs in KMA were included and the distribution was found to be approximately proportionate to the total population of the LGAs.

### 3.2 Characteristics of study subjects

The demographic characteristics of the participants from both surveys are shown in Table 1. Two younger age groups from the 2013 survey and both age groups from the 2014 survey have been analyzed in greater detail: Nutritional indices and data on the number of OPV doses received are shown only for these age groups.

There was a small imbalance in the proportion of male to female infants in the 6–9 month age group, with 60% and 55% of the subjects being male in the 2013 and 2014 surveys respectively. Subsequent analyses showed that there was no difference in seroprevalence levels between males and females and that gender was not a significant predictor of seropositivity.

### 3.3 Seroprevalence in 2013

Overall seroprevalence was low in the 6–9 month age group. The seroprevalence levels plateaued in the 36–47 month age group; with only minor gains in the 5–9 year and 10–14 year age groups (Table 2).

Among subjects aged 6–9 months, seroprevalence was 58% (95% confidence interval [CI] 51–66%) to poliovirus type 1, 42% (95% CI 34–49%) to poliovirus type 2, and 52% (95% CI 44–60%) to poliovirus type 3. Among subjects aged 36–47 months, the seroprevalence was 93% (95% CI 88–96%) to poliovirus type 1, 85% (95% CI 78–90%) for poliovirus type 2, and 87% (95% CI 81–92%) to poliovirus type 3. Seroprevalence was significantly higher in the 36–47 months group for each type as compared to 6–9 months group (p < 0.0001 for all three serotypes).

### 3.4 Trends in seroprevalence over time

Table 3 shows the seroprevalence and 95% confidence intervals for the 6–9 month age group in 2013 and 2014 serosurveys. The seroprevalence levels in this age group improved in 2014, from 58% in 2013 to 72% (95% CI 65–79%) in 2014 for type 1 (p = 0.009), from 42% to 59% (95% CI 52–66%) for type 2 (p = 0.001), and from 52% to 65% (95% CI 57–72%) for type 3 (p = 0.016).

**Fig. 1:**
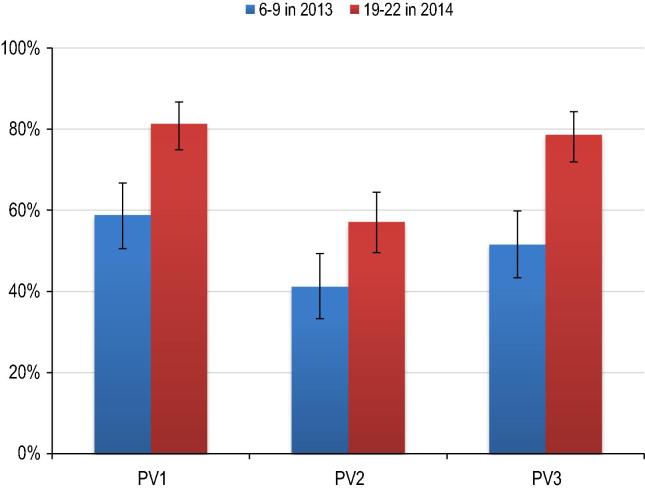
Seroprevalence and 95% confidence intervals, by poliovirus serotype in 6–9 month old cohort in 2013 and 19–22 month old cohort in 2014.

Comparison of the seroprevalence levels for the 6–9 month cohort of 2013 becoming 19–22 months old in the 2014 survey; is shown in Fig. 1. An increase in seroprevalence to the tune of 23%, 16% and 27% was seen for the three poliovirus types: from 58% for infants 6–9 months old in 2013 to 81% for children 19–22 months old in 2014 (95% CI 75–87%) for type 1 (p < 0.001), from 41% to 57% (95% CI 50–64%) for type 2 (p = 0.004), and from 52% to 79% (95% CI 72–84%) for type 3 (p < 0.001). Even in 2014, type 2 seroprevalence remained low (approximately 60%), and was similar for both the 6–9 month and 19–22 month age groups.

### 3.5 Risk factors

Table 3 shows the analysis of seroprevalence by demographic characteristics and OPV doses for 6–9 month infants in the 2013 and 2014 surveys. In both surveys, seroprevalence for all three serotypes is associated with the number of routine doses received by the participants (p = 0.021, <0.0001, 0.048 in 2013 and p < 0.001 for all three types in 2014). Seroprevalence is associated with the total number of OPV doses received for types 1 and 3 in the 2013 survey (p = 0.002 and 0.008) and with all three types in 2014 (p < 0.001, =0.002, <0.001 respectively). There is an association with the number of SIA OPV doses for types 1 and 3 in 2014 (p = 0.036 and 0.013). A similar trend is seen in 2013 but does not attain significance.

In the 36–47 months age group in 2013, there was significant association between seropositivity to all three serotypes with the number of RI OPV doses received (p = 0.002, 0.02, and 0.02 for types 1, 2 and 3, respectively) and for type 1 with the SIA doses (p < 0.0001) and total OPV doses (p < 0.0001). For type 2, there was also a significant association between mother’s educational status and seropositivity (p = 0.03) possibly due to better routine immunization in the educated class. For the 19–22 month old age group in 2014, seropositivity is significantly associated with routine OPV doses for types 2 and 3 (p < 0.001 and p = 0.031), and with SIA doses and total doses for types 1 and 3 (p < 0.001 for all tests), possibly due to bOPV contributing heavily to SIA and total doses.

The logistic regression analysis is shown in Table 4. In 2013, receiving at least one SIA dose was a significant predictor of seropositivity for types 1 and 3: children who received at least one SIA dose had nearly three-fold increased odds of being seropositive for both types (type 1 OR = 2.77, 95% CI 1.08–7.49; type 3 OR = 2.86, 95% CI 1.14–7.68). For type 2, RI doses were a significant predictor of seropositivity: each additional dose increased the odds of seropositivity by 2.22 (95% CI 1.66, 3.12). In 2014, receiving an SIA dose was a significant predictor only for type 3, but showed a similar three-fold increased odds of seropositivity (OR = 3.20, 95% CI 1.32–7-76). However, RI doses were a significant predictor for all three types, with each additional RI dose conferring a 50–60% increase in odds of seropositivity (type 1 OR = 1.60, 95% CI 1.29–1.99; type 2 OR = 1.64, 95% CI 1.33–2.02; type 3 OR = 1.49, 95% CI 1.21–1.83).

The results indicate that receiving OPV doses remains a significant predictor of seropositivity. There is no consistent relationship between seropositivity and socioeconomic and nutritional parameters across the two surveys.

## 4 Discussion

These seroprevalence surveys document the immunity profiles in the historical polio reservoir area of northern Nigeria over a crucial period before and after the interruption of poliovirus transmission. Our surveys provide several new insights.

The 2013 survey showed that seroprevalence was unexpectedly low in the 6–9 months age group. Nevertheless, we do believe that low seroprevalence in this age group had reflected recent programmatic gaps. Attacks on health care workers had resulted in cancellation of the March 2013 SIA campaign in Kano and could potentially have affected the quality of the subsequent campaigns. The seroprevalence in 36–47 month children was high and there were only minor gains in seroprevalence levels in the older age groups of 5–9 and 10–14 years. Nevertheless, both in 6–9 month and 36–47 month age groups, an increasing number of OPV doses resulted in increasing levels of seroprevalence. This confirms the effectiveness of OPV in inducing immunity in these populations.

While seroprevalence recovered to an extent in 6–9 month age group by 2014, it remained below 75% for all three serotypes. There was significant improvement in the 19–22 month age group over the corresponding 6–9 month group of 2013. Besides age and number of vaccine doses, seroprevalence has a reasonable consistent relationship with vaccine types used in routine and supplementary immunization, like impact of bOPV SIA doses on type 1 and 3 immunity and that of tOPV RI doses on type 2. Continued low seroprevalence to type 2 remains a key concern and indicates less exposure to type 2 containing vaccines. It seems improvement in program quality and incremental gains in immunity helped Kano achieve polio free status and no wild virus or VDPV has been reported from the state in 2015 and till date in 2016.

While the exact threshold immunity to interrupt transmission is not known for northern Nigeria, seroprevalence in Kano, Nigeria is well below the levels reported from Egypt and India (98–99%) in the final stages of eradication [21], [22]. Although such extremely high immunity levels were probably not needed in the Nigerian context to interrupt transmission, the data does suggest that further increase in vaccination coverage in both routine and SIAs must be achieved to improve and sustain high immunity levels.

Our study had limitations, mainly associated with facility-based design. This study was not population-based and thus the results are not generalizable to the entire population. Nevertheless, our survey demonstrated that in the study population who accessed care in a general government hospital, the seroprevalence rates were suboptimal. It is probably safe to assume that the seroprevalence levels could be even lower in other populations without access to health services. For data collection, we had to rely on parental recall for a number of variables, including vaccination history, maternal education, and age. In addition, the study design and sample size were not meant to be able to assess differences in small sub-populations.

Though the community-level acceptance for vaccination was variable in the area, we observed a good parental response for participation in the seroprevalence survey in the health facility-based surveys. We plan to repeat seroprevalence surveys in Kano Nigeria to monitor the population immunity and measure the impact of tOPV-bOPV switch and introduction of IPV in the routine immunization program.

## Funding

World Health Organization (using a Grant from Rotary International).

## Ethical approvals

Research Ethics Committee of Aminu Kano Teaching Hospital and Ethical Review Committee of World Health Organization, Geneva.

## Conflict of interest

No authors reported a conflict of interest.

The findings and conclusions in this report are those of the authors and do not necessarily represent the official position of the Centers for Disease Control and Prevention or other collaborating agencies.

## Figures and Tables

**Table 1:** Demographic and other attributes of study population, Kano Metropolitan Area, Northern Nigeria, 2013 and 2014.

	2013								2014			
	6–9 months		36–47 months		5–9 years		10–14 years		6–9 months		19–22 months	
	N	%	N	%	N	%	N	%	N	%	N	%
Total	154		143		151		154		183		184	

Gender												
Female	61	39.6	71	49.7	61	40.4	77	50.0	82	44.8	93	50.5
Male	93	60.4	72	50.4	90	59.6	77	50.0	101	55.2	91	49.5

Mother’s education												
Primary or less	78	50.7	81	56.6	97	64.2	99	64.3	110	60.1	111	60.3
Secondary or more	75	48.7	62	43.4	54	35.8	55	35.7	73	39.9	73	39.7
Unknown	1	0.7	0	0.0	0	0.0	0	0.0	0	0.0	0	0.0

Father’s education												
Primary or less	35	22.7	30	21.0	41	27.2	51	33.1	46	25.1	51	27.7
Secondary or more	118	76.6	111	77.6	109	72.2	102	66.2	133	72.7	130	70.7
Unknown	1	0.7	2	1.4	1	0.7	1	0.7	4	2.2	3	1.6

Children in household												
0	0	0.0	0	0.0	24	15.9	47	30.5	0	0.0	0	0.0
1–2	130	84.4	108	75.5	101	66.9	77	50.0	143	78.1	157	85.3
More than 2	24	15.6	35	24.5	26	17.2	30	19.5	34	18.6	24	13.0
Unknown	0	0.0	0	0.0	0	0.0	0	0.0	6	3.3	3	1.6

Wasting												
No	108	70.1	116	81.1					148	80.9	129	70.1
Moderate	23	14.9	21	14.7					24	13.1	34	18.5
Severe	18	11.7	4	2.8					11	6.0	20	10.9
Unknown	5	3.3	2	1.4					0	0.0	1	0.5

Stunting												
No	107	69.5	98	68.5					161	88.0	110	59.8
Moderate	21	13.6	28	19.6					16	8.7	39	21.2
Severe	23	14.9	15	10.5					6	3.3	34	18.5
Unknown	3	2.0	2	1.4					6	0.0	1	0.5

Routine OPV doses												
0	32	20.8	16	11.2					41	22.4	39	21.2
1	18	11.7	13	9.1					14	7.7	8	4.4
2	8	5.2	21	14.7					18	9.8	17	9.2
3	42	27.3	36	25.2					20	10.9	14	7.6
4	51	33.1	51	35.7					90	49.2	103	56.0
Unknown	3	2.0	6	4.2					0	0.0	3	1.6

SIA OPV doses												
0	28	18.2	5	3.5					28	15.3	9	4.9
1–3	51	33.1	20	14.0					58	31.7	16	8.7
4–6	61	39.6	30	21.0					68	37.2	27	14.7
7+	10	6.5	79	55.2					26	14.2	125	67.9
Unknown	4	2.6	9	6.3					3	1.6	7	3.8

Total OPV doses												
0	9	5.8	3	2.1					5	2.7	3	1.6
1–3	29	18.8	6	4.2					36	19.7	7	3.8
4–6	47	30.5	22	15.4					54	29.5	19	10.3
7+	62	40.3	101	70.6					85	46.5	147	79.9
Unknown	7	4.6	11	7.7					3	1.6	8	4.4

**Table 2:** Poliovirus seroprevalence for different age groups, Kano, Nigeria, 2013.

Age group	Number of subjects	Type 1 (%)	Type 2 (%)	Type 3 (%)
6–9 months	154	58.4	41.6	51.9
36–47 months	143	93.0	84.6	87.4
5–9 years	151	94.0	92.7	89.4
10–14 years	154	96.1	92.9	89.0

Total	**602**	**85.2**	**77.7**	**79.2**

**Table 3:** Seroprevalence among subjects aged 6–9 months, by demographic or other attributes, Kano Metropolitan Area, Northern Nigeria, 2013, 2014.

	2013							2014						
	No. of children	PV1		PV2		PV3		No. of children	P1		P2		P3	
		n	%	n	%	n	%		n	%	n	%	n	%
Total	154	90	58.4	64	41.6	80	52.0	181	131	72.4	107	59.1	117	64.6

Gender^a^														
Female	61	36	59.0	26	42.6	33	54.1	81	54	66.7	49	60.5	47	58.0
Male	93	54	58.1	38	40.9	47	50.5	100	77	77.0	58	58.0	70	70.0

Mother’s education^a^														
Primary or less	78	**38**	**48.7**	**23**	**29.5**	**34**	**43.6**	109	78	71.2	59	54.1	68	62.4
Secondary or more	75	**51**	**68.0**	**40**	**53.3**	**46**	**61.3**	72	53	73.6	48	66.7	49	68.1

Father’s education^a^														
Primary or less	35	18	51.4	**7**	**20.0**	14	40.0	46	31	67.4	23	50.0	28	60.9
Secondary or more	118	71	60.2	**56**	**47.5**	66	55.9	131	97	74.0	83	63.3	86	65.6

Children in household^a^														
1–2	130	77	59.2	55	42.3	**73**	**56.2**	141	101	71.6	87	61.7	92	65.3
More than 2	24	13	54.2	9	37.5	**7**	**29.2**	34	25	73.5	16	47.1	23	67.7

Wasting^b^														
No	108	62	57.4	45	41.7	55	50.9	146	101	69.2	82	56.1	93	63.7
Moderate	23	14	60.9	9	39.1	11	47.8	24	21	87.5	20	83.3	18	75.0
Severe	18	11	61.1	8	44.4	10	55.6	11	9	81.2	5	45.5	6	54.5

Stunting^b^														
No	107	**69**	**64.5**	48	44.9	**61**	**57.0**	159	113	71.1	94	59.1	105	66.0
Moderate	21	**10**	**47.6**	10	47.6	**8**	**38.1**	16	13	81.2	10	62.5	10	62.5
Severe	23	**9**	**39.1**	5	21.7	**8**	**34.8**	6	5	83.3	3	50.0	2	33.3

Routine OPV doses^b^														
0	32	**14**	**43.8**	**3**	**9.4**	**12**	**37.5**	41	**18**	**43.9**	**11**	**26.8**	**13**	**31.7**
1	18	**9**	**50.0**	**4**	**22.2**	**9**	**50.0**	13	**9**	**69.2**	**6**	**46.2**	**10**	**76.9**
2	8	**4**	**50.0**	**3**	**37.5**	**4**	**50.0**	18	**16**	**88.9**	**11**	**61.1**	**13**	**72.2**
3	42	**27**	**64.3**	**21**	**50.0**	**22**	**52.4**	20	**14**	**70.0**	**13**	**65.0**	**13**	**65.0**
4	51	**34**	**66.7**	**32**	**62.8**	**31**	**60.8**	89	**74**	**83.1**	**66**	**74.2**	**68**	**76.4**

SIA OPV doses^b^														
0	28	10	35.7	13	46.4	9	32.1	28	**19**	**67.9**	15	53.6	**13**	**46.4**
1–3	51	32	62.8	20	39.2	28	54.9	57	**37**	**64.9**	35	61.4	**38**	**66.7**
4–6	61	39	63.9	27	44.3	35	57.4	67	**51**	**76.1**	42	62.7	**43**	**64.2**
7+	10	6	60.0	4	40.0	6	60.0	26	**23**	**88.5**	15	57.7	**22**	**84.6**

Total OPV doses^b^														
0	9	**1**	**11.1**			**0**	**0.0**	5	2	**40.0**			**0**	**0.0**
1–3	29	**12**	**41.4**			**13**	**44.8**	36	20	**57.1**			**16**	**45.7**
4–6	47	**32**	**68.1**			**27**	**57.5**	55	37	**68.5**			**37**	**68.5**
7+	62	**40**	**64.5**			**36**	**58.1**	84	71	**84.5**			**63**	**75.0**

Covariates significantly associated with seropositivity at α = 0.05 are indicated in **bold**.

a Pearson Chi-square test.

b Cochrane-Armitage test for trend.

**Table 4:** Adjusted odds ratios and 95% confidence intervals for seroprevalence by RI and SIA doses received in children 6–9 months of age.

	Comparison	2013			2014		
		Odds ratio	95% CI	Type III p-value	Odds ratio	95% CI	Type III p-value
*Type 1*							
RI dose (continuous)	Each additional dose	1.08	[0.85, 1.37]	0.523	**1.60**	[1.29, 1.99]	<0.001
Any campaign dose	1+ vs. 0 SIA doses	**2.77**	[1.08, 7.49]	0.037	1.55	[0.60, 4.00]	0.366

*Type 2*^a^							
RI dose (continuous)	Each additional dose	**2.22**	[1.66, 3.12]	<0.001	**1.64**	[1.33, 2.02]	<0.001

*Type 3*							
RI dose (continuous)	Each additional dose	1.22	[0.98, 1.54]	0.082	**1.49**	[1.21, 1.83]	<0.001
Any campaign dose	1+ vs. 0 SIA doses	**2.86**	[1.14, 7.68]	0.030	**3.20**	[1.32, 7.76]	0.01

a Analysis of campaign doses was excluded for Type 2, as children in the 6–9 month age group only received Type 2 containing vaccine through routine immunization in 2013 (all relevant campaign doses were bOPV), and were exposed to at most one tOPV campaign dose in 2014.
